# Effect of Berberine on Atherosclerosis and Gut Microbiota Modulation and Their Correlation in High-Fat Diet-Fed ApoE−/− Mice

**DOI:** 10.3389/fphar.2020.00223

**Published:** 2020-03-13

**Authors:** Min Wu, Shengjie Yang, Songzi Wang, Yu Cao, Ran Zhao, Xinye Li, Yanwei Xing, Longtao Liu

**Affiliations:** ^1^Guang’anmen Hospital, China Academy of Chinese Medical Sciences, Beijing, China; ^2^Xiyuan Hospital, China Academy of Chinese Medical Sciences, Beijing, China; ^3^Beijing University of Chinese Medicine, Beijing, China

**Keywords:** berberine, atherosclerosis, gut microbiota, inflammation, lipid metabolism

## Abstract

Atherosclerosis and its associated cardiovascular diseases (CVDs) are serious threats to human health and have been reported to be associated with the gut microbiota. Recently, the role of berberine (BBR) in atherosclerosis and gut microbiota has begun to be appreciated. The purposes of this study were to observe the effects of high or low doses of BBR on atherosclerosis and gut microbiota modulation, and to explore their correlation in ApoE^–/–^ mice fed a high-fat diet. A significant decrease in atherosclerotic lesions was observed after treatment with BBR, with the effect of the high dose being more obvious. Both BBR treatments significantly reduced total cholesterol, APOB100, and very low-density lipoprotein cholesterol levels but levels of high/low-density lipoprotein cholesterol and lipoprotein (a) were only reduced by high-dose BBR. Decreased pro-inflammatory cytokines tumor necrosis factor-alpha, interleukin (IL)-1β, IL-6 and increased anti-inflammatory IL-10 and adiponectin levels were observed in the high-dose BBR group, but no decrease in IL-6 or increase in IL-10 was evident using the low-dose of BBR. 16S rRNA sequencing showed that BBR significantly altered the community compositional structure of gut microbiota. Specifically, BBR enriched the abundance of *Roseburia*, *Blautia*, *Allobaculum*, *Alistipes*, and *Turicibacter*, and changed the abundance of *Bilophila*. These microbiota displayed good anti-inflammatory effects related to the production of short-chain fatty acids (SCFAs) and were related to glucolipid metabolism. *Alistipes* and *Roseburia* were significantly enriched in high-dose BBR group while *Blautia* and *Allobaculum* were more enriched in low-dose, and *Turicibacter* was enriched in both BBR doses. Metagenomic analysis further showed an elevated potential for lipid and glycan metabolism and synthesis of SCFAs, as well as reduced potential of TMAO production after BBR treatment. The findings demonstrate that both high and low-dose BBR can improve serum lipid and systemic inflammation levels, and alleviate atherosclerosis induced by high-fat diet in ApoE^–/–^ mice. The effects are more pronounced for the high dose. This anti-atherosclerotic effect of BBR may be partly attributed to changes in composition and functions of gut microbiota which may be associated with anti-inflammatory and metabolism of glucose and lipid. Notably, gut microbiota alterations showed different sensitivity to BBR dose.

## Introduction

Atherosclerosis is the common pathological basis of many cardiovascular diseases (CVDs). Although lipid-lowering drugs, interventional therapy, and other conventional treatments have been widely used to reduce the threats to human health, atherosclerosis and its associated CVDs are still the leading cause of death worldwide (Anderson et al., 1991; Vilahur et al., 2014). Atherosclerosis is accompanied by dyslipidemias, including elevated levels of low-density lipoprotein cholesterol (LDL-C), total cholesterol (TC), triglyceride (TG), etc. (Ju et al., 2018). Statins are the first-line pharmacotherapy for dyslipidemias and effectively control lipid levels and reduce major adverse cardiovascular events (MACE) (Catapano et al., 2016; Kobiyama and Ley, 2018). However, statins have been reported to be correlated with various adverse events related to myopathy, renal disease, hepatobiliary disorders, and other events (Law and Rudnicka, 2006). In addition, atherosclerosis is considered to be a chronic inflammatory disease of the large and medium arteries (Ross, 1999; Libby, 2002; Hansson, 2005), with changes in pro-inflammatory cytokines such as tumor necrosis factor-alpha (TNF-α),interleukin (IL)-6, and IL-1β, and anti-inflammatory cytokines including IL-10 (Tedgui and Mallat, 2006). The role of these cytokines in the development, progression, and complications of atherosclerosis has been reviewed (Tedgui and Mallat, 2006).

Studies in the past decade have shown that the gut microbiota is associated with certain human diseases, including obesity (Boulange et al., 2016), type 2 diabetes (Qin et al., 2012), hypercholesterolemia (Martinez et al., 2009) and CVDs (Tang et al., 2017), all of which are associated with atherosclerosis (Jonsson and Backhed, 2017). The role of the gut microbiota in atherosclerosis has begun to be appreciated in recent years, mainly including the regulation of inflammation and immunity, and cholesterol and lipid metabolism by gut microbiota and its metabolites (Jonsson and Backhed, 2017; Ma and Li, 2018). These findings have highlighted the great potential preventative and therapeutic benefits for atherosclerosis that might be realized by targeting the gut microbiome.

Berberine (BBR) is an isoquinoline alkaloid that can be isolated from various medicinal plants, such as *Coptis chinensis* Franch and *Cortex phellodendri*. BBR has various biological functions including anti-inflammatory activity, improvement of cholesterol levels and lipid metabolism, and prevention of metabolic diseases. These functions are also very important in anti-atherosclerosis effect. Nutraceuticals containing BBR can significantly reduce plasma LDL-C levels of elderly statin-intolerant hypercholesterolemic patients (Marazzi et al., 2011). Another study showed that when it plus low-dose statins and/or ezetimibe, most coronary artery disease (CHD) patients who were high-dose statin intolerant could achieve target LDL-C levels within 3 to 6 months (Marazzi et al., 2019). Of note, the therapeutic effects of BBR on these inflammatory and metabolic diseases, such as emergent mild diarrhea (Yue et al., 2019), ulcerative colitis (Cui et al., 2018), obesity (Xie et al., 2011; Zhang et al., 2015), hyperlipidemia (Wang et al., 2017) and diabetes (Zhang et al., 2012), appeared to be related to the regulation of the gut microbiota.

Previous studies have indicated that the anti-atherosclerotic effect of BBR may be associated with changes in the gut microbiota, specifically the increased abundance of *Akkermansia*, altered abundance of Firmicutes and Verrucomicrobia, and decreased serum trimethylamine *N*-oxide (TMAO) levels (Zhu et al., 2018; Shi et al., 2018). However, the specific linking of the role of BBR in gut microbiota with atherosclerosis and the potential mechanism are still unknown. Therefore, we investigated the correlation of the effect of BBR (high and low doses) on gut microbiota with the alleviation of atherosclerosis, including improvements in plaque area, serum lipid levels, and systemic inflammation. The results of 16S rRNA sequencing and metagenomic analysis showed that the anti-atherosclerotic effect of high and low-dose BBR may be partly attributed to the changes in composition and functions of the gut microbiota, which may be related to anti-inflammatory and the metabolism of glucose and lipid.

## Materials and Methods

### Animal Model

All animal experimental procedures conformed to the ARRIVE guidelines, the United States National Institutes of Health Laboratory Animals Care and Use guidelines, and the Peking University Animal Investigation Committee guidelines. Six-week-old male ApoE^–/–^ mice and C57BL/6J mice were purchased from the Jackson Laboratory (Bar Harbor, ME, United States) and were raised at the animal laboratory center of Peking University. The mice were housed in a specific pathogen-free environment with a room temperature at 22–24°C, relative humidity of 50%, 12 h light/dark cycle, and freely acquirable food and water. The ApoE^–/–^ mice were fed with HFD containing 21% saturated fat and 0.15% cholesterol (Beijing Keao Xieli Feed Co., Ltd., Beijing, China) for 13 weeks. C57BL/6J mice (*n* = 12) were fed a normal-chow diet as the Control group. Mice fed with HFD were randomly divided into three groups (*n* = 12 in each group). The Model group was treated with 0.9% sterile saline by gavage once daily. The High group was given 100 mg/kg body weight BBR hydrochloride (97% purity, Xi’an Platt Biological Engineering Co., Ltd., Xi’an, China) by gavage once daily. The Low group was given 50 mg/kg body weight BBR by gavage once daily. Berberine were administrated for 13 weeks. C57BL/6J mice were administered with 0.9% sterile saline by gavage once daily. The experiment lasted 26 weeks.

### Plaque Lesion Analysis

Heart tissues containing the aortic arch were excised from the proximal aortic root to the iliac artery branch and placed in a 4% paraformaldehyde environment for 6 h. The atherosclerotic lesion area of aorta was evaluated by *en face* Oil red O staining as previously described (Shi et al., 2018). Measurement of the lesion size was based on hematoxylin and eosin staining of sections of paraffin embedded aortic root. The area and size of the lesion were analyzed by a blind observer using Image J software.

### Measurement of Serum Lipid Profiles

Blood samples were taken from the retro-orbital plexus after overnight fasting and centrifuged, and the serum was immediately stored at −80°C. Measurements of the levels of TC, TG, LDL-C, high density lipoprotein cholesterol (HDL-C), very LDL-C (VLDL-C), lipoprotein (a) [Lp (a)], Apolipoprotein B100 (ApoB100) and Apolipoprotein A-1 (APOA-1) were made using enzymatic methods in a model AD2700 automated biochemical analyzer (Olympus, Tokyo, Japan).

### Measurement of Inflammatory Cytokines

The levels of serum inflammatory cytokines, including TNF-α, IL-1β, IL-6, IL-10, and adiponectin (ADPN), were measured using ELISA kits (Abcam, Cambridge, United Kingdom) according to the manufacturer’s instructions. The level of high-sensitivity C-reactive protein (Hs-CRP) was measured by a nephelometry immunoassay.

### Fecal DNA Extraction and PCR Amplification

Total genomic DNA was extracted from fecal samples using Omega Stool DNA Kit (Omega Bio-Tek, Norcross, GA, United States) following the manufacturer’s instructions. The extracted fecal DNA was used as template. Amplification of the V3-V4 hypervariable regions of 16S rRNA gene was performed with the forward primer 338F(5′-ACTCCTACGGGAGGCAGCAG-3′) and reverse primer 806R(5′-GGACTACNNGGG TATCTAAT-3′). The PCR reaction system (total 25 μL) included 12.5 μL KAPA 2G Robust HotStart Ready Mix (Kapa Biosystems, Wilmington, MA, United States), 1 μL forward and 1 μL reverse primers (5 μM), 5 μL DNA template (30 ng), and 5.5 μL double-distilled water. The amplification procedure involved an initial denaturation at 95°C for 5 min, 28 cycles of denaturation at 95°C for 45 s, 50 s annealing at 55°C, 45 s elongation at 72°C, final extension at 72°C for 10 min, and storage at 4°C. PCR products were detected by 1% agarose gel electrophoresis to determine the size of the amplified target band and purified using Agencourt AMPure XP kit (Beckman Coulter, Brea, CA, United States). The library quality was assessed on the Qubit Fluorometer (Life Technologies, Carlsbad, CA, United States) and the Bioanalyzer 2100 system (Agilent, Santa Clara, CA, United States). Sequencing was performed on Illumina Miseq PE300 (Illumina Inc., San Diego, CA, United States) at Allwegene Technology Co., Ltd. (Beijing, China). All raw sequences have been submitted to the Sequences Read Archive database at the NCBI under accession number SRP226688.

### High-Throughput Sequencing and Bioinformatics Analysis

USEARCH (Edgar, 2010), QIIME v.1.9.1 (Caporaso et al., 2010) and VSEARCH (Rognes et al., 2016) software were used to process the 16S rRNA gene sequence. Paired-end Illumina reads were quality controlled by FastQC v.0.11.5 (Andrews, 2010), and USEARCH was used to join the paired-end reads and rename. Removal of barcodes and primers, and quality filter were performed and non-redundancy reads were removed. All 16S rRNA chimeric sequences were detected and removed using the UCHIME algorithm against the RDP Gold database (Edgar, 2013). The non-chimeric sequences were sorted by abundance, and clustered into operational taxonomic units (OTUs) using the UNOISE algorithm (100% similarity threshold) with low abundant sequences removed (eight sequences). The OTU table was produced using VSEARCH software and the representative sequence of each OTU was classified by RDP classifier algorithm against the SILVA128 database with a confidence threshold of 70%.

Alpha (α) diversity analysis, including the Shannon index and Chao1 index, were carried out using the vegan (Oksanen et al., 2007) package in R. The beta (β) diversity was evaluated by Principal Coordinate Analysis (PCoA) based on the Bray Curtis distance algorithm. The LEfSe algorithm was used to identify microbes that differed significantly among groups at different taxonomy levels, with a linear discriminant analysis (LDA) score threshold of 4. The Statistical Analysis of Metagenomic Profiles (STAMP) software was used to analyze significant taxa between different groups using Welch’s *t*-test with 0.95 confidence interval permutations (Parks et al., 2014). Differentially abundant OTUs were detected using the EdgeR package and depicted as volcano plots. Representative sequences from most abundant OTUs (relative abundance > 0.002) were selected to make a maximum likelihood phylogenetic tree (ML tree) using IQ-Tree software (Nguyen et al., 2015) with the following parameters: model gtr, 5000 Ultrafast bootstrap and 5000 SH-like approximate likelihood ratio test. Then, the tree was visualized by iTOL web software (Letunic and Bork, 2019) with annotations of relative abundance of groups^1^. Potential correlation between gut microbiota and serum levels of lipids and inflammatory cytokines was evaluated based on the Spearman rank correlation. Analysis and plotting were done using the vegan package.

### Library Construction and Metagenomic Sequencing

The qualified DNA extract was broken to an average fragment of 300 bp size approximately with a Covaris S220 (Covaris, Inc., MS, United States) for paired-end library construction through NEBNext Ultra II DNA Library Prep Kit (NEB, Beijing, China). Paired-end sequencing was conducted on Illumina HiSeq4000 (Illumina Inc., San Diego, CA, United States) utilizing HiSeq 4000 Reagent Kits v2 under the manufacturer’s instructions.

### Sequence Quality Control and Genome Assembly

SeqPrep^2^ was used to separate the adapter sequences from the 3′ and 5′ end of paired-end reads. Low-quality reads with insufficient length or quality value were eliminated with Sickle^3^. Reads were aligned to the host DNA sequences using the BWA^4^, which removed contaminated reads with high alignment similarity. MEGAHIT (Li et al., 2015) was used to assemble the metagenomics data, and contigs which were over 300 bp in length were used as the final assembled result.

### Gene Prediction, Taxonomy, and Functional Annotation

MetaGene (Noguchi et al., 2006) was employed on predicting open reading frames (ORFs) of the assembled contigs. Predicted ORFs which were over 100 bp in length were translated into amino acid sequences according to the translation table of the National Center for Biotechnology Information (NCBI).

All predicted genes were clustered using the CD-HIT (Fu et al., 2012) (parameters: 95% identity, 90% coverage). The representative sequences were selected from the longest sequences in each cluster to construct the non-redundant gene catalog. SOAPaligner (Li et al., 2008) was used to map the reads after quality control to the non-redundant gene catalog with 95% identity, and the gene abundance in the corresponding samples was evaluated.

Non-redundant gene catalog representative sequences were aligned to the NCBI NR database (truncating e-value to 1e^–5^) through BLASTP (Version 2.2.28 +) (Altschul et al., 1997) and further obtained taxonomic annotation. BLASTP against the Kyoto Encyclopedia of Genes and Genomes (KEGG) database was performed to determine the KEGG annotation. KOBAS 2.0 was used for functional annotation based on the comparison results.

### Statistical Analyses

Data are expressed as mean ± SEM. Part of the 16S rRNA analysis was carried out in R software. In addition, differential levels of atherosclerotic plaques, lipids, inflammatory cytokines, and the abundance of potential gut biomarkers at the genus level were tested using one-way analysis of variance (ANOVA), followed by the Sidak multiple comparisons test. The Kruskal-Wallis rank sum test was used to test differential abundance of functional genus and KEGG pathways. A *p*-value < 0.05 was considered statistically significant. GraphPad Prism 8.0 (GraphPad, La Jolla, CA, United States) was used for the statistical analysis.

## Results

### BBR Attenuates HFD-Induced Atherosclerosis

**FIGURE 1:**
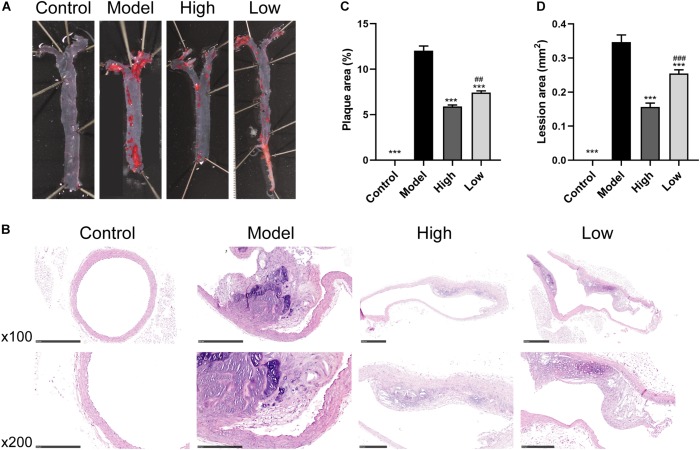
Berberine attenuates HFD-induced atherosclerosis. **(A)** Representative images of *en face* Oil Red O staining of aorta. **(B)** H&E staining of aortic root. **(C** and **D)** Quantitative analysis of plaque area in aorta and lesion size in the aortic root (*n* = 6 per group). Data are expressed as mean ± SEM. Differences were assessed by one-way ANOVA. ^∗∗∗^ *p* < 0.001, vs Model; ^##^ *p* < 0.01, ^###^ *p* < 0.001, High vs Low.

Oil Red O and H&E staining showed that the atherosclerotic plaques and lesions in the Model group were higher than those in the Control group (Figures 1A,B). However, significant reductions in atherosclerotic lesions after BBR treatment were observed in both the High and Low groups. Quantitative analysis also showed that atherosclerotic plaque area and lesion size decreased significantly in both the High and Low groups compared with the Model group (both *p* < 0.001). The decrease was more significant in the High group compared to the Low group (*p* < 0.05 for atherosclerotic plaque area and *p* < 0.01 for lesion size) (Figures 1C,D). These data suggested that HFD feeding markedly promoted the development of atherosclerosis, while the high or low dose of BBR could ameliorate HFD-induced atherosclerosis, with the effect of high dose being more pronounced.

### BBR Ameliorates Serum Lipid Levels in HFD-Fed ApoE−/− Mice

**FIGURE 2:**
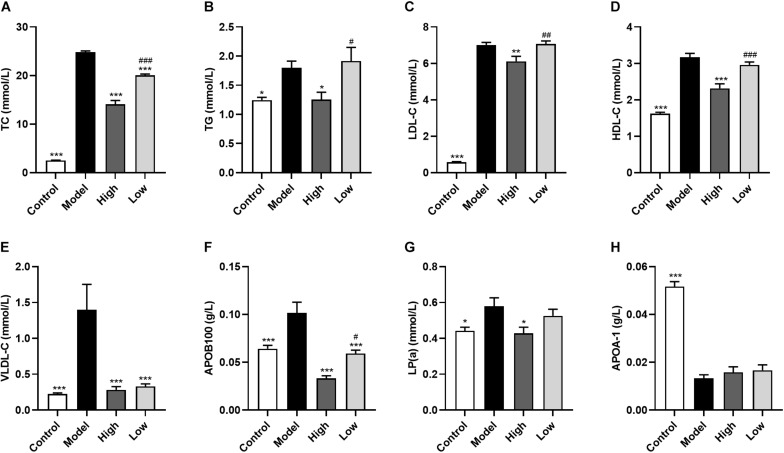
Berberine ameliorates serum lipid levels in HFD-fed ApoE^–/–^ mice. **(A)** Total cholesterol, **(B)** triglyceride, **(C)** low-density lipoprotein cholesterol, **(D)** high density lipoprotein cholesterol, **(E)** very low-density lipoprotein cholesterol, **(F)** lipoprotein (a), **(G)** Apolipoprotein B100, and **(H)** Apolipoprotein A-1. Data are expressed as mean ± SEM (*n* = 12). Differences among the groups were assessed by one-way ANOVA. ^∗^ *p* < 0.05, ^∗∗^ *p* < 0.01, ^∗∗∗^ *p* < 0.001, vs Model; ^#^ *p* < 0.05, ^##^ *p* < 0.01, ^###^ *p* < 0.01, High vs Low.

Model group mice had significantly increased serum levels of TC (*p* < 0.001), TG (*p* < 0.05), HDL-C (*p* < 0.001), LDL-C (*p* < 0.001), APOB100 (*p* < 0.001), LP(a) (*p* < 0.05), and VLDL-C (*p* < 0.001), and significantly decreased APOA-1 levels (*p* < 0.001) compared with the Control group mice. In addition, both high and low doses of BBR significantly reduced TC, APOB100, and VLDL-C levels (all *p* < 0.001), with the decrease of TC (*p* < 0.001) and APOB100 (*p* < 0.05) being significantly different between the High and Low groups. TG (*p* < 0.05), HDL-C (*p* < 0.001), LDL-C (*p* < 0.01), and LP(a) (*p* < 0.05) were only significantly reduced in the High group compared with the Model group. However, there was no significant difference in serum APOA-1 levels after treatment with high or low doses of BBR (*p* > 0.05) (Figure 2).

### BBR Improves Systemic Inflammation

**FIGURE 3:**
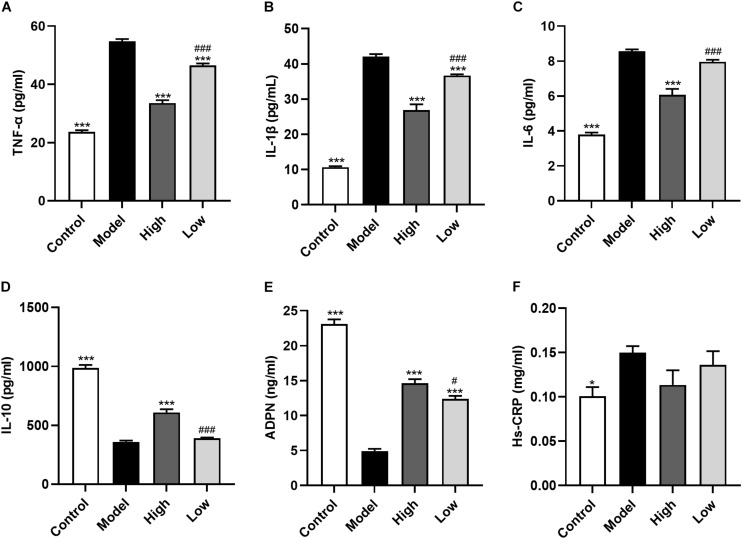
Berberine improves systemic inflammation. **(A-F)** Serum levels of TNF-α, IL-1β, IL-6, Hs-CRP, IL-10, and ADPN. Data are expressed as mean ± SEM (*n* = 12). Differences among the groups were assessed by one-way ANOVA. ^∗^ *p* < 0.05, ^∗∗∗^ *p* < 0.001, vs Model; ^#^ *p* < 0.05, ^###^ *p* < 0.001, High vs Low.

Atherosclerosis is an inflammatory disease and inflammatory cytokines play an important role in the process of atherosclerosis. Here, we found that compared with the Control group mice, serum levels of TNF-α, IL-1β, IL-6 were significantly increased, and IL-10 and ADPN levels were significantly decreased in HFD-induced atherosclerosis mice (Model group; all *p* < 0.001). These changes could be reversed by BBR treatment. High dose BBR significantly reduced the levels of TNF-α, IL-1β, and IL-6, and significantly increased levels of IL-10 and ADPN (all *p* < 0.001). However, in the Low group, only TNF-α and IL-1β levels were decreased and ADPN levels were increased (all *p* < 0.001), while the decrease in IL-6 and increase in IL-10 levels were not significant (*p* > 0.05). Moreover, the levels of these inflammatory cytokines were significantly different between the High and Low groups (*p* < 0.05 or *p* < 0.001). Serum Hs-CRP level was higher in the Model group than in the Control group (*p* < 0.05), while there was no significant difference between the other groups (*p* > 0.05) (Figure 3).

### Effect of BBR on Composition of Gut Microbiota

To determine the effects of BBR on gut microbiota composition, the Illumina Miseq PE300 high-throughput sequencing platform was used to conduct 16S rRNA amplicon paired-end sequencing. After sequence quality control and denoising, a total of 1,027,879 high-quality sequences were obtained from 30 samples. After removal of singleton (> 8) and non-bacteria sequences (such as mitochondria), 1085 OTUs were obtained with an average of 25,801 sequences per sample (range: 21,577-46,467), accounting for 75.3% of the total sequences.

**FIGURE 4:**
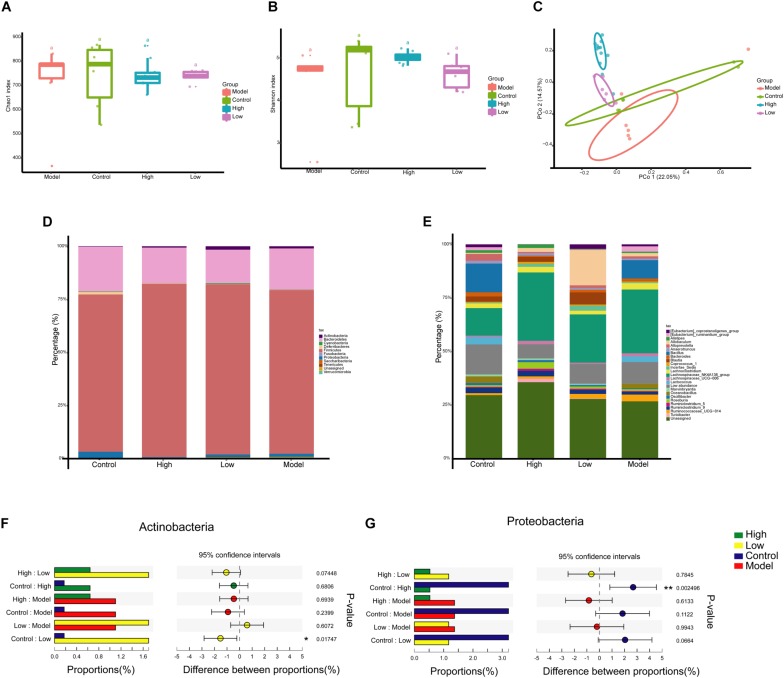
Berberine significantly alters the composition of gut microbiota in HFD-induced atherosclerosis mice. **(A** and **B)** Diversity of the gut microbiome assessed by Chao1 and Shannon indices among the four groups. **(C)** Principal Coordinate Analysis (PCoA) based on the Bray Curtis distance algorithm. **(D)** Taxonomic composition of each group at the phylum level. **(E)** Taxonomic composition at the genus level. **(F** and **G)** Relative abundance of Proteobacteria and Actinobacteria in the one-way ANOVA bar plot on the phylum level. ^∗^ *p* < 0.05, ^∗∗^ *p* < 0.01.

The OTU table was normalized to the minimum number of reads (21,577) per sample and then diversity comparison was performed. Alpha diversity analysis revealed no significant difference in gut microbiota diversity between each group based on Chao 1 and Shannon indices (*p* > 0.05) (Figures 4A,B). PCoA based on Bray Curtis distance was used to visualize the dissimilarity in the compositions of the bacterial communities. The community compositional structure differed in the four groups, among which the High and Model groups and the Low and Model groups were significantly different (*p* < 0.001) (Figure 4C). A BBR dose-related difference was observed on PC2, in which the High group clustered separately from the Low group, and this explained 14.57% of the total variation in microbial composition. Mice treated with high or low doses of BBR showed similar microbial characteristics on PC1, which explained 22.05% of the total variation (Figure 4C).

Next, we investigated the differences of gut microbiota at different taxonomic levels. The compositions of gut microbiota at the phylum and genus levels in each group are shown in Figures 4D,E. At the phylum level, Firmicutes (75.4%) and Bacteroidetes (15.1%) were the two most dominant phyla, followed by Proteobacteria (3.3%) and Actinobacteria (2.5%) (Figure 4D). However, the abundance of these major phyla did not appear to differ significantly between the groups. One-way ANOVA analysis showed that only Proteobacteria and Actinobacteria had significant differences in abundance between the Control group and the High or Low group (*p* < 0.05), with decreased Proteobacteria abundance and increased Actinobacteria abundance after BBR treatment (Figures 4F,G).

We extracted all the representative sequences from OTUs with relative abundant > 0.2%. In total, 116 bacterial OTUs were selected and all the representative sequences were used to make an ML tree (Supplementary Figure S1). There were 116 OTUs annotated at the genus level, with color ranges identifying the phylum to which the OTUs belonged. These OTUs mainly distributed in Firmicutes and Bacteroidetes phylum, and *Lachnospiraceae_NK4A136_group* and unassigned genus. Heatmap was used to annotate the relative abundance of OTUs, and the OTUs with higher abundance were mainly distributed in *Allobaculum*, *Bacillus*, *Lachnospiraceae_NK4A136_group*, *Blautia*, etc. The outermost layer was added with circle annotation at the family level.

**FIGURE 5:**
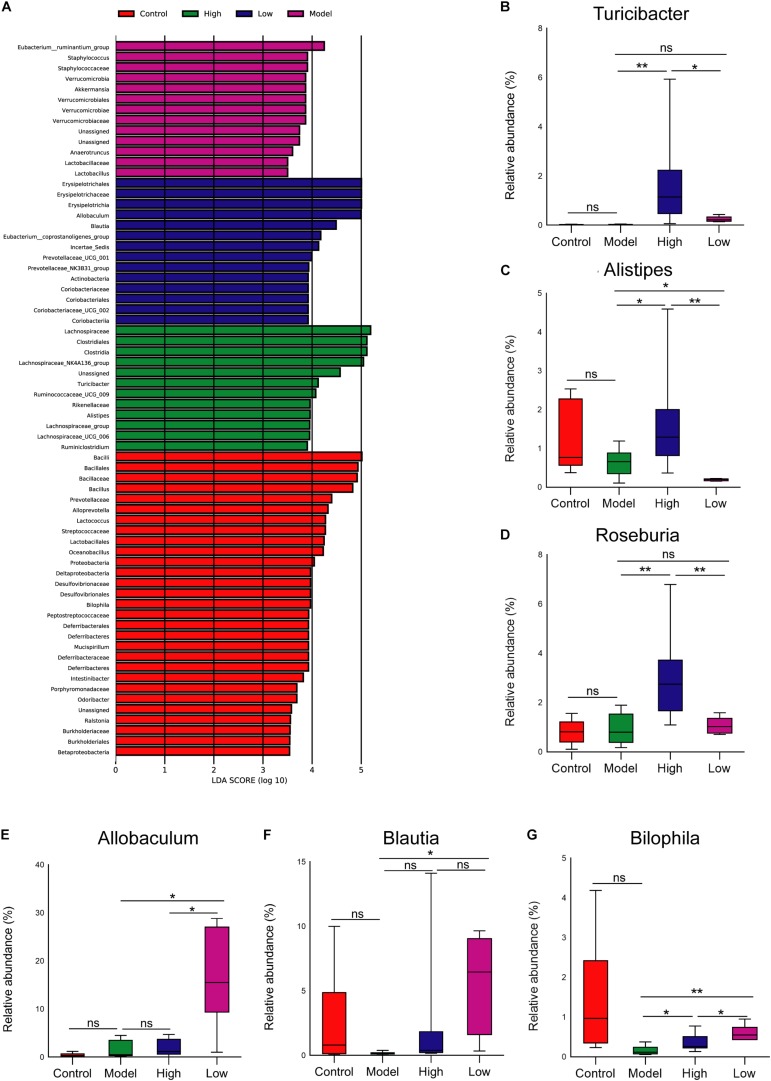
**(A)** Gut microbiota comparisons among the four groups analyzed by LEfSe at different taxonomy levels, with taxa meeting LDA score threshold > 4 being listed. **(B-F)** The relative abundance of **(B)** *Turicibacter*, **(C)** *Alistipes*, **(D)** *Roseburia*, **(E)** *Allobaculum*, **(F)** *Blautia*, and **(G)** *Bilophila* at the genus level. ^∗^ *p* < 0.05, ^∗∗^ *p* < 0.01; ns, not significant.

At the genus level, LEfSe analysis showed that *Turicibacter* and *Alistipes* were enriched in the High group, *Allobaculum* and *Blautia* were enriched in the Low group, and the abundance of *Bilophila* was relatively higher in the Control group than that in other groups (Figure 5A). Moreover, STAMP analysis revealed significant differences in *Roseburia* between the groups. Further analysis of the relative abundance of microbes that differed among groups revealed that the abundance of *Turicibacter* was higher, and *Bilophila* and *Blautia* were lower in the Model group than that in the Control group, but these differences were not significant (*p* > 0.05). Both high and low doses of BBR increased the abundance of *Turicibacter*, and it was more obvious and significant at the high dose (*p* < 0.05) (Figure 5B). The abundance of *Alistipes* and *Roseburia* was only significantly enriched in the High group (*p* < 0.05 and < 0.01, respectively; Figures 5C,D). Of note, compared with the Model group, the abundance of both *Allobaculum* and *Blautia* was significantly increased when treated with low-dose BBR (both *p* < 0.05), but there was no significant change after high-dose BBR treatment (*p* > 0.05) (Figures 5E,F). In addition, the abundance of *Bilophila* decreased in the High group (*p* < 0.05) and increased in the Low group (*p* < 0.01) compared with the Model group (Figure 5G). These results showed that BBR significantly altered the composition of gut microbiota in HFD-induced atherosclerosis mice, and different doses of BBR showed different influence on specific microbes.

To further analyze these differences more precisely at the OTU level, we analyzed the number of significantly enriched and depleted OTUs in each group (Supplementary Figure S2). Compared with the Model group, 80 OTUs were enriched in the Control group, 213 OTUs were enriched in the Low group, and 228 OTUs were enriched in the High group. The number of OTUs with a significant difference increased obviously after BBR treatment, revealing an increasing change.

### Correlation Between Gut Microbiota and Lipid Profile and Inflammatory Cytokines

**FIGURE 6:**
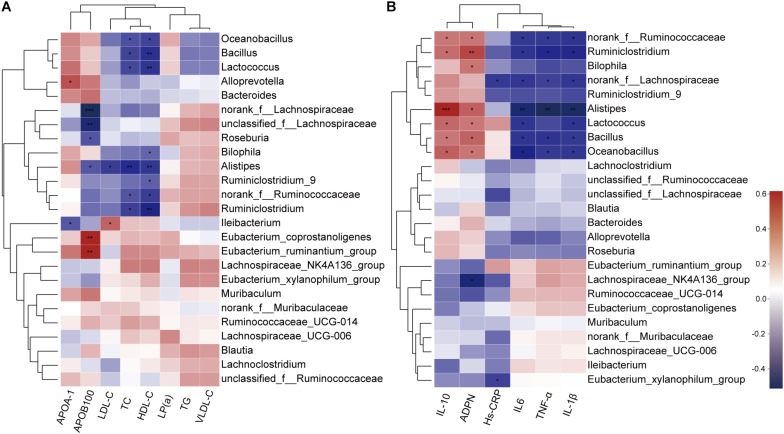
Spearman rank correlations between gut microbiota abundance and serum levels of lipids **(A)** and inflammatory cytokines **(B)**. Red indicate being positive correlation and blue being negative. * 0.01 < *p* ≤ 0.05, ** 0.001 < *p* ≤ 0.01, *** *p* ≤ 0.001.

As BBR significantly altered the composition of the gut microbiota and improved serum lipid levels and systemic inflammation in HFD-induced atherosclerotic mice, we further analyzed the correlation between gut microbiota and serum levels of lipids and inflammatory cytokines using the Spearman rank correlation. We observed a significant negative correlation between serum levels of TC, HDL-C, LDL-C, APOB100 and the abundance of gut microbiota (at the genus level, in the top 25 genera), among which *Alistipes* showed the most significant negative correlation with lipid levels. Other potential genus-level biomarkers including *Roseburia* and *Bilophila* were also negatively correlated (Figure 6A). In addition, the anti-inflammatory cytokines IL-10 and ADPN displayed a positive correlation with gut microbiota, and pro-inflammatory cytokines IL-1β, IL-6, TNF-α and Hs-CRP were negatively correlated (Figure 6B). Notably, the correlation with *Alistipes* was still very significant.

### Functional Alterations in Gut Microbiota Induced by BBR

**FIGURE 7:**
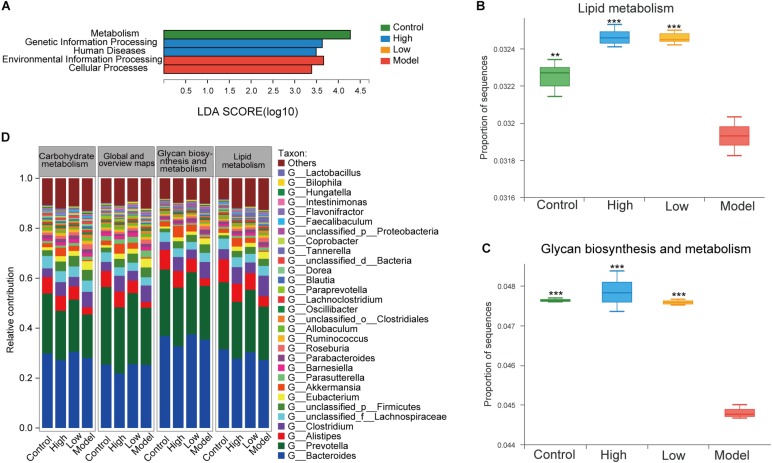
**(A)** LEfSe analysis of KEGG pathways comparisons at levels 1 (LDA > 2.5, P < 0.05). **(B)** Differential abundance of lipid metabolism pathways. **(C)** Differential abundance of glycan biosynthesis and metabolism pathways. **(D)** The top 25 contributing species for functional genes related to metabolism at level 2. Differential abundance of KEGG pathways were assessed by Kruskal-Wallis rank sum test. ** *p* < 0.01, *** *p* < 0.001, vs Model.

To further investigate the effect of BBR on the functional changes of the gut microbiome in HFD-induced atherosclerotic mice, we determined the extent of different KEGG pathways and genes enriched in each group (three mice per group). LEfSe analysis was performed to detect the KEGG pathways with significantly different abundance between the four groups. At KEGG level 1, environmental information processing and cellular processes were significantly enriched in the model group, while the proportion of sequences related to metabolism was remarkably reduced (LDA > 2.5, *p* < 0.05, Figure 7A). This also supports the view that atherosclerosis is a disease that is closely associated with a variety of metabolic and cellular processes (Jie et al., 2017). At level 2, lipid metabolism, glycan biosynthesis and metabolism and carbohydrate metabolism are important components under the metabolism classification. The samples from the model group displayed reduced potential for lipid metabolism (*p* < 0.01) and glycan biosynthesis and metabolism (*p* < 0.001) compared with controls, while these were both increased after treatment with high or low dose BBR (all *p* < 0.001, Figures 7B,C). Differences between the high and low dose BBR groups were not significant. In addition, there was no significant difference in carbohydrate metabolism between groups (*p* > 0.05). Analysis of the relative contribution of species (at the genus level) to functional attributes found that (Figure 7D) *Bacteroides*, *Prevotella* and *Alistipes* were the major contributors to these functions. Consistent with the analysis results of the 16S rRNA sequencing, a lower abundance of *Alistipes* displayed a decreased functional contribution in the model group compared with the control group, and the functional contribution accordingly increased after high and low dose BBR treatment, with a higher abundance of *Alistipes*. Other potential genera that could be classified as gut biomarkers included *Roseburia*, *Allobaculum*, *Blautia*, and *Bilophila*; all of these were in the top thirty enriched genera with important contributions to lipid and glucose metabolism.

**FIGURE 8:**
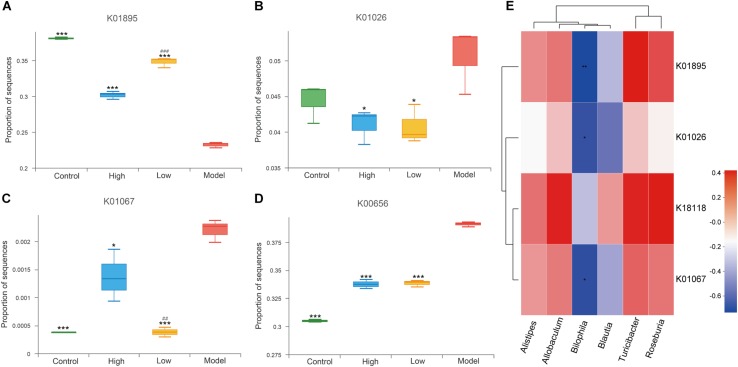
**(A–D)** Differential enrichment of specific gut microbial KOs for SCFAs production among four groups. * *p* < 0.05, *** *p* < 0.001, vs Model. ^###^ *p* < 0.001, High vs Low. **(E)** Spearman rank correlations between abundance of six biomarkers at genus level and SCFAs producing-related KOs. Red indicate being positive correlation and blue being negative.

Short-chain fatty acids (SCFAs) are important metabolites of gut microbiota (den Besten et al., 2013) and have been reported to be closely related to inflammatory defense and anti-atherosclerosis (Ohira et al., 2017; Bultman, 2018). Sequences of SCFA-producing enzymes and associated enzymes identified by reference to the gut microbiota gene catalog were determined as previously described (Claesson et al., 2012; Jie et al., 2017). We screened the metagenomic data of enzymes related to the production of acetate (acetyl-CoA synthetase, K01895), propionate (propionyl-CoA: succinate-CoA transferase, K18118; propionate CoA transferase, K01026), and butyrate (acetyl-CoA hydrolase, K01067). Butyryl-CoA transferase was not successfully matched. We found that, compared with the model group, acetyl-CoA synthetase (K01895) was significantly enriched in the high and low dose BBR groups (all *p* < 0.001), indicating increased synthesis potential of acetate. This was more significant in the low dose BBR group than in the high (*p* < 0.001, Figure 8A). However, the abundance of K01026 was reduced in both the high and low dose BBR groups, which exhibited reduced potential for the synthesis of propionate compared with the model group (*p* < 0.05, Figure 8B). Another related gene, K18118, showed no significant difference between the groups (*p* > 0.05). Butyrate is produced through the condensation of two acetyl-CoA molecules and then reduction to butyryl-CoA, which is subsequently converted into butyrate via the classical pathway (Koh et al., 2016). Acetyl-CoA hydrolase(K01067) was observed to be more enriched in the model group compared with the high-dose (*p* < 0.05) and low-dose BBR groups (*p* < 0.001), suggesting a lower potential for butyrate synthesis in the model group (Figure 8C). In addition, pyruvate-formate lyase(K00656), a key enzyme in the biosynthesis of formate (Leonhartsberger et al., 2002), showed higher enrichment in the model group than in the BBR groups (all *p* < 0.001), which suggested that the gut microbiota in mice with atherosclerosis might produce more formate (Figure 8D). Previous studies have reported that formate was associated with hypertension and might be implicated in other atherosclerotic cardiovascular disease (ACVD)-related functions (Holmes et al., 2008; Goodrich et al., 2016). Spearman rank correlations (Figure 8E) showed that Alistipes, *Allobaculum, Blautia, Turicibacter*, and *Roseburia* exhibited a positive correlation with KEGG orthologues (KOs) related to SCFA production, while *Bilophila* exhibited a significant negative correlation with SCFA production (*p* < 0.05 or *p* < 0.01).

**FIGURE 9:**
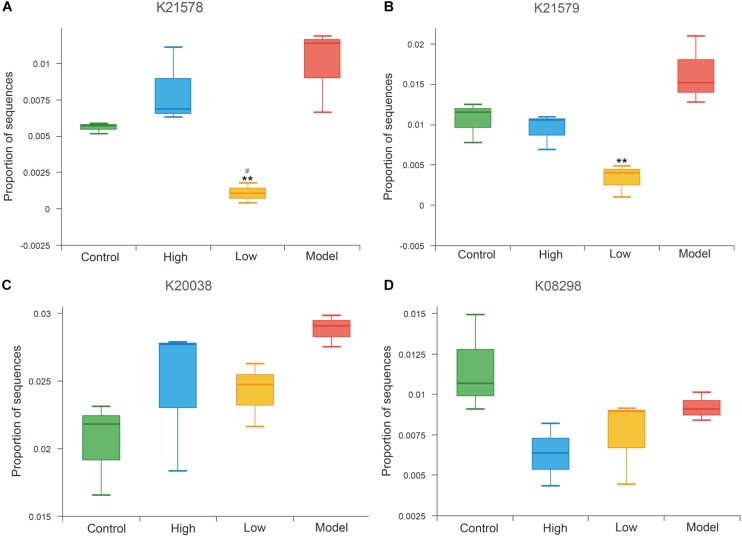
Abundance differences in specific gut microbial KOs for TMAO production. ** *p* < 0.01, vs Model. ^#^ *p* < 0.05, High vs Low.

Trimethylamine N-oxide is an important pro-atherogenic metabolite produced by gut microbiota, and gut microbial enzymes were involved in the formation of its precursor trimethylamine (TMA) (Wang et al., 2011; Koeth et al., 2013). Previous studies confirmed that choline TMA lyase (K20038), betaine reductase (K21578, K21579) and *L*-carnitine CoA-transferase (K08298) are the main TMA-lyases involved in TMA production (Fennema et al., 2016; Zeisel and Warrier, 2017; Janeiro et al., 2018). Results at the KO level exhibited decreased abundance of betaine reductase (K21578, K21579) in both the high and low dose BBR groups compared to the model group, and was especially significantly in the low dose group (all *p* < 0.01, Figures 9A,B). The abundance of choline TMA lyase (K20038) and L-carnitine CoA-transferase (K08298) were also reduced after high or low dose BBR treatment, but were not significant (*p* > 0.05, Figures 9C,D).

## Discussion

In this study, we investigated the effect of BBR on atherosclerosis in HFD-fed ApoE^–/–^ mice. We observed the changes in serum levels of lipid and inflammatory cytokines as well as compositional structure and functions of gut microbiota after treatment with different doses of BBR, and correlated these changes with atherosclerosis. BBR treatment attenuated HFD-induced atherosclerosis, decreased atherosclerotic plaques and lesion areas, and ameliorated the level of serum lipids and inflammatory cytokines. The effects of the high dose of BBR were more significant than the effects of the low dose. 16S rRNA amplicon sequencing showed that BBR significantly altered the community compositional structure of the gut microbiota, and different doses of BBR showed different changes. Further analysis of environmental factor and metagenomic function displayed the effects of BBR on inflammation, glucose and lipid metabolism, and TMAO production in gut microbiota.

Elevated lipid levels are a major risk factor for atherosclerotic CVDs. Since Kong et al. (2004) identified BBR as a new cholesterol-lowering drug (Kong et al., 2004), the therapeutic effect of BBR on dyslipidemia has been confirmed and further explored in recent years (Kong et al., 2008; Zhang et al., 2008). Studies performed in dyslipidemia patients and hyperlipidemic mice showed that BBR significantly reduced serum TC, TG, and LDL-C levels (Kong et al., 2004; Zhang et al., 2008). In addition, these results were supported by two meta-analyses (Lan et al., 2015; Ju et al., 2018), which also reported increased HDL-C levels after BBR treatment. The current results demonstrated that both high and low doses of BBR significantly reduced the levels of TC, APOB100, and VLDL-C, while TG, HDL-C, LDL-C and LP (a) levels were reduced only in the High group. Our results do not indicate an increase in HDL-C after BBR treatment. APOA-1 has an anti-atherogenic effect, and injection of APOA-1 can reduce intracellular cholesterol levels in ApoE^–/–^ mice (Pourcet and Staels, 2016; Gaddis et al., 2018). However, there was no significant increase in reduced APOA-1 levels in the atherosclerosis mice using the high or low dose of BBR in our study.

Atherosclerosis is a chronic inflammatory disease, and inflammation is constantly induced throughout the course of the disease (Libby, 2002; Hansson, 2005; Tedgui and Mallat, 2006). The anti-inflammatory effects of BBR, such as suppressing the expression of pro-inflammatory genes and improving HFD-induced systemic inflammation, have been widely reported (Jeong et al., 2009; Zhang et al., 2012). Previous studies in HFD-induced atherosclerosis mice reported that BBR administration remarkably reduced the mRNA expression of pro-inflammatory cytokines, including TNF-α, IL-1β, and IL-6, in ileal and carotid arteries (Shi et al., 2018; Zhu et al., 2018). Consistent with these results, serum levels of TNF-α, IL-1β, and IL-6 were significantly decreased by the high dose of BBR, while in the low dose group, only TNF-α and IL-1β levels were decreased. In addition, anti-inflammatory cytokines IL-10 (Han and Boisvert, 2015; Metghalchi et al., 2015) and insulin sensitizing hormone ADPN (Lin et al., 2015) have been shown to have anti-atherosclerosis effects. The production of IL-10 was increased in a mouse model of colitis induced by dextran sulfate sodium after BBR administration (Hong et al., 2012). Presently, ADPN levels increased significantly after both high and low dose BBR treatment compared to the Model group, but IL-10 levels were increased only by the high dose of BBR. Combined with previous reports, the present observations support the view that the alleviation of inflammation may be an important mechanism for the reduction of atherosclerosis in BBR-treated HFD-fed mice.

The role of the gut microbiota in atherosclerosis has begun to be studied in recent years. Multiple studies have identified a strong link between the gut microbiota and atherosclerosis (Law and Rudnicka, 2006; Catapano et al., 2016; Ju et al., 2018; Kobiyama and Ley, 2018). To explore the relationship between the anti-atherosclerosis effect of BBR and gut microbiota, 16S rRNA sequencing was used to observe the changes of gut microbiota in each group. Alpha diversity analysis did not reveal a significant decrease in fecal microbial diversity after BBR treatment, which is inconsistent with other research results (Zhang et al., 2015; Zhu et al., 2018). However, in our study beta diversity analysis showed that the community compositional structure was significantly different after treatment with the high or low dose of BBR, which do support the findings of a previous study (Shi et al., 2018). Presently, Firmicutes (75.4%) and Bacteroidetes (15.1%) are the two most dominant phylum at the phylum level, accounting for 90.5%. This was similar to the composition of human gut microbiota in prior studies, with Bacteroidetes and Firmicutes constituting more than 90% of the taxa (Qin et al., 2010; The Human Microbiome Project Consortium, 2012). In our study, the higher abundance was followed by Proteobacteria (3.3%) and Actinobacteria (2.5%), which is inconsistent with the prior finding of Proteobacteria and Verrucomicrobia (Shi et al., 2018). In addition, only decreased Proteobacteria abundance in High group and increased Actinobacteria abundance in Low group was significantly observed after BBR treatment compared with the Control group. These observations might be explained by the fact that, although the species abundance or number of the microbiota varied between individuals, the gut microbiota was semblable among individuals at higher taxonomic levels, such as the phylum level (Jonsson and Backhed, 2017).

At the genus level, LEfSe and STAMP analyses revealed significant differences between groups. Previous studies have shown that animal-based diets and HFD increases the abundance of *Alistipes* (David et al., 2014; Wan et al., 2019). Moreover, *Alistipes* has demonstrated a good anti-inflammatory effect in human and animal experiments. *Alistipes* is an obvious microbial characteristic of patients with inflammatory bowel disease (Schirmer et al., 2018) and increased *Alistipes* has been directly related to the improvement of colitis induced by dextran sulfate sodium in mice (Dziarski et al., 2016). In particular, a significant correlation was described between acetic acid production and the relative abundance of *Alistipes* (Yin et al., 2018). In addition, the genus *Roseburia* was reported to produce butyric acid and metabolize dietary plant polysaccharides, and its abundance was decreased in animal-based diets (David et al., 2014; Kim et al., 2018). In our study, the abundance of *Alistipes* was also increased in the HFD-fed mice compared with the Control group, but not significantly, while no significant change was observed in the abundance of *Roseburia*. The high dose of BBR resulted in the significant increase in the abundance of *Alistipes* and *Roseburia*, which may be beneficial in the reduction of inflammation in BBR. Butyric acid and acetic acid are both SCFAs, and the role of SCFAs in relieving inflammation has been increasingly reported. For example, increased intake of SCFAs was proven to be beneficial in the treatment of colitis (Harig et al., 1989), and the anti-inflammation effect might be mediated by G-protein coupled receptor 43 (Maslowski et al., 2009). The SCFA-producing genera *Allobaculum* and *Blautia* were significantly enriched and systemic inflammation decreased in BBR-treated HFD-fed rats (Zhang et al., 2012). Our study supported the latter finding. However, it is worth noting that treatment with the low dose of BBR significantly increased the abundance of *Blautia* and *Allobaculum*, while the high dose of BBR did not.

*Turicibacter* is a genus that is associated with disorders of glucose and lipid metabolism, and is negatively correlated with random blood glucose in diabetic fatty rats (Lai et al., 2018; Zhou et al., 2019). *Turicibacter* has also been associated with intestinal butyric acid (Zhong et al., 2015), which could increase the secretion and sensitivity of insulin, and has significant anti-obesity and anti-inflammatory effects (Gao et al., 2009; Li et al., 2013; De Vadder et al., 2014). A meta-analysis showed that the decrease of *Turicibacter* is consistent with increased inflammation in obesity (Jiao et al., 2018). In addition, the decrease in *Turicibacter* has been associated with abundant dietary cholesterol and dietary fat (Everard et al., 2014; Zhong et al., 2015; Dimova et al., 2017). We found that both high and low doses of BBR enriched the abundance of *Turicibacter*, and the effect was significant and more pronounced at the high dose. This suggests that the anti-atherosclerosis effect may be related to the interference of BBR on glycolipid metabolism-related gut microbiota. Notably, the abundance of *Bilophila* decreased in HFD-fed mice, contrary to the prior finding that animal-based diets increased the abundance of *Bilophila* (David et al., 2014). *Bilophila* reportedly acts synergistically with HFD to promote higher inflammation and bile acid dysmetabolism, resulting in more serious glucose dysmetabolism and hepatic steatosis (David et al., 2014; Natividad et al., 2018). Presently, the abundance of *Bilophila* decreased in the high-dose BBR group and increased in the low-dose BBR group compared with the Model group. However, compared with normal control mice, the abundance of *Bilophila* was decreased after BBR treatment, although it was not obvious.

Environmental factor analysis helped us further determine the potential correlation between lipids, inflammatory cytokines, and the gut microbiota. In addition, metagenomic analysis based on functional changes of the gut microbiome showed that the potential for lipid metabolism and glycan biosynthesis and metabolism was elevated in atherosclerotic model mice after high or low dose BBR treatment. The analysis of species and functional contribution further highlighted the importance of these genus-level biomarkers for glucose and lipid metabolism, among which *Alistipes* contributed the most. SCFAs are known to be defensive players for inflammation and atherosclerosis (Ohira et al., 2017) and we observed an elevated potential for the synthesis of acetate and butyrate in both high and low-dose BBR groups compared to atherosclerosis model mice. Notably, the alteration of acetate was more significant in the low dose BBR group. However, the synthesis potential of propionate seemed to be decreased after BBR treatment, which was not consistent with the protective effects of propionate on hypertension, cardiovascular damage and anti-atherosclerosis (Bultman, 2018; Bartolomaeus et al., 2019). A heatmap showed that *Alistipes*, *Allobaculum*, *Blautia*, *Turicibacter* and *Roseburia* were positively correlated with SCFA production, while *Bilophila* was significantly negatively correlated. This supported the results that *Bilophila* acted synergistically with HFD to promote higher inflammation and more serious glucose dysmetabolism (David et al., 2014; Natividad et al., 2018). Consistently, the abundance of *Bilophila* decreased in the high-dose BBR group in our study. In addition, compared with the atherosclerotic mice, the abundance of TMAO-related enzymes was reduced in both the high and low dose BBR group, and betaine reductase decreased reduced more significantly in the low-dose BBR group. These results showed that different doses of BBR might have different inhibitory effects on TMAO production.

Our results suggested that high and low-dose BBR induced the alteration of abundance and function in certain gut microbiota that may be related to inflammation and glucose and lipid metabolism. These findings partially support the view that BBR may at least partly improve systemic inflammation and lipid level by regulating gut bacteria related to inflammation and glucolipid metabolism, thereby reducing HFD-induced atherosclerosis. Indeed, evidence is accumulating that gut microbiota and its metabolites, such as SCFAs, TMAO, and secondary bile acids, play a role in atherosclerosis by regulating inflammation and the metabolism of lipid, cholesterol and glucose (Barrington and Lusis, 2017; Jonsson and Backhed, 2017; Kasahara et al., 2017; Ma and Li, 2018). SCFAs have anti-atherogenic effect, in addition, it can inhibit the fat synthesis in enterocytes and adipocytes, and suppress biosynthesis of cholesterol and LDL formation in the liver, besides their anti-inflammatory effects (Chistiakov et al., 2015). The involvement of SCFAs in atherosclerosis has been reviewed (Brown and Hazen, 2018). The proatherogenic metabolite TMAO has been associated with the increased risk of atherosclerosis (Wang et al., 2011; Tang et al., 2013). Elevated TMAO levels contributed to foam cell formation and platelet hyperreactivity and inhibited reverse cholesterol transport in macrophages (Wang et al., 2011; Koeth et al., 2013; Zhu et al., 2016). Notably, previous study has shown that BBR treatment can significantly reduce serum TMAO levels (Shi et al., 2018).

Our study has some limitations. Considering the fact that sample quantity is relatively small, the persuasiveness of the results is limited. Although we explained the relationship between the regulation effect of BBR on gut microbiota and the reduction of atherosclerosis based on 16S rRNA sequencing and metagenomic analysis, further fecal bacteria transfer experiments are still needed to clarify the direct link of BBR on gut microbiota with atherosclerosis. This study was conducted in ApoE^–/–^ mice fed with HFD. In the future study, we should extend to clinical atherosclerosis patients to further well explore the relationship of effect of BBR on gut microbiota and atherosclerosis.

Nonetheless, our study increases the accumulating evidence that BBR can protect against atherosclerosis induced by HFD. Previous studies have shown that BBR could effectively reduce plasma LDL-C levels in patients with CHD and/or hypercholesterolemia who were statin intolerant (Marazzi et al., 2011, 2019). Studies performed in hyperlipidemia mice and meta-analyses also supported that BBR improved dyslipidemia (Lan et al., 2015; Ju et al., 2018). In addition, the anti-inflammatory effects of BBR, including the reduction of HFD-induced systemic inflammation and the suppression of proinflammatory gene expression, have been widely confirmed (Jeong et al., 2009; Zhang et al., 2012). However, the mechanism of these effects is still unclear due to the low BBR absorption rate. Recent studies suggested that the anti-atherosclerotic effect of BBR may be related to changes in gut microbiota, but the specific association and potential mechanism are still unknown (Shi et al., 2018; Zhu et al., 2018). The present study may provide a novel explanation. Our study ulteriorly supports the anti-atherogenic effect of BBR by targeting the gut microbiota at both the species and function levels. In some extent, this suggests the potential of BBR and certain gut microbiome as an alternative or adjuvant treatment strategy for atherosclerotic patients who are intolerant to conventional drugs (such as statins) or under an increased risk of adverse effects.

## Conclusion

In conclusion, both high and low dose BBR can improve serum lipid and inflammation levels and alleviate atherosclerosis in a mouse model of HFD-induced atherosclerosis. This anti-atherosclerotic effect of BBR may be partly attributed to changes in composition and functions of gut microbiota which may be related to anti-inflammatory activity and the metabolism of glucose and lipid. In addition, different doses of BBR had different effects, among which the attenuation of atherosclerosis was more obvious at the high dose of BBR, but the alterations of composition and functions in gut microbiota showed different sensitivity to BBR dose.

## Data Availability Statement

The datasets generated for this study can be found in the Sequences Read Archive database at the NCBI under accession number PRJNA579048.

## Ethics Statement

The animal study was reviewed and approved by the Animal Investigation Committee of Peking University.

## Author Contributions

MW, SY, LL and YX designed the experiment and wrote the manuscript. SW and YC aided in the design of the illustrations. RZ and XL commented on the manuscript. All authors approved the manuscript for publication.

## Conflict of Interest

The authors declare that the research was conducted in the absence of any commercial or financial relationships that could be construed as a potential conflict of interest.

## Footnotes

1 http://itol.embl.de

2 https://github.com/jstjohn/SeqPrep

3 https://github.com/najoshi/sickle

4 http://bio-bwa.sourceforge.net

## Supplementary Material

FIGURE S1 Most abundant OTU members of the gut bacterial microbiome (Top 116).

FIGURE S2 Differentially enriched and depleted OTUs between each group compared with the Model group. **(A)** Control vs Model; **(B)** Low vs Model; **(C)** High vs Model. Each point in the figure represents an individual OTU, of which the red points for significant enriched OTUs, green points for significant depleted OTUs, gray points for OTUs with no significant difference. The number labeled in the figure is the number of OTUs that significantly enriched or decreased.
