# Supplementary figures and images for: Effect of Berberine on Atherosclerosis and Gut Microbiota Modulation and Their Correlation in High-Fat Diet-Fed ApoE−/− Mice
Source: Front Pharmacol. 2020 Mar 13;11:223. doi: 10.3389/fphar.2020.00223 (PMC7083141; doi:10.3389/fphar.2020.00223)

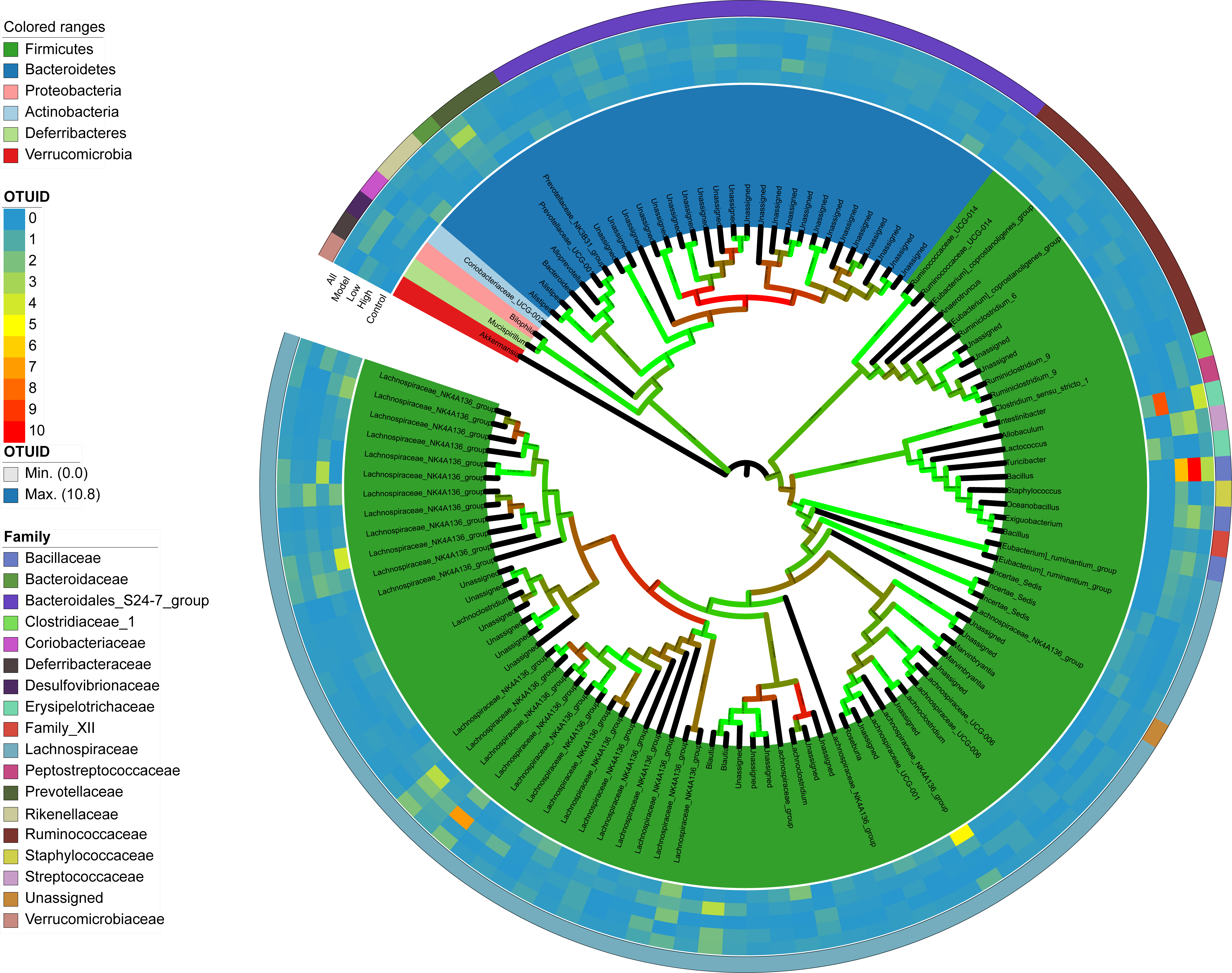

Supplement: FIGURE S1 — Most abundant OTU members of the gut bacterial microbiome (Top 116). [file Image_1.TIF]

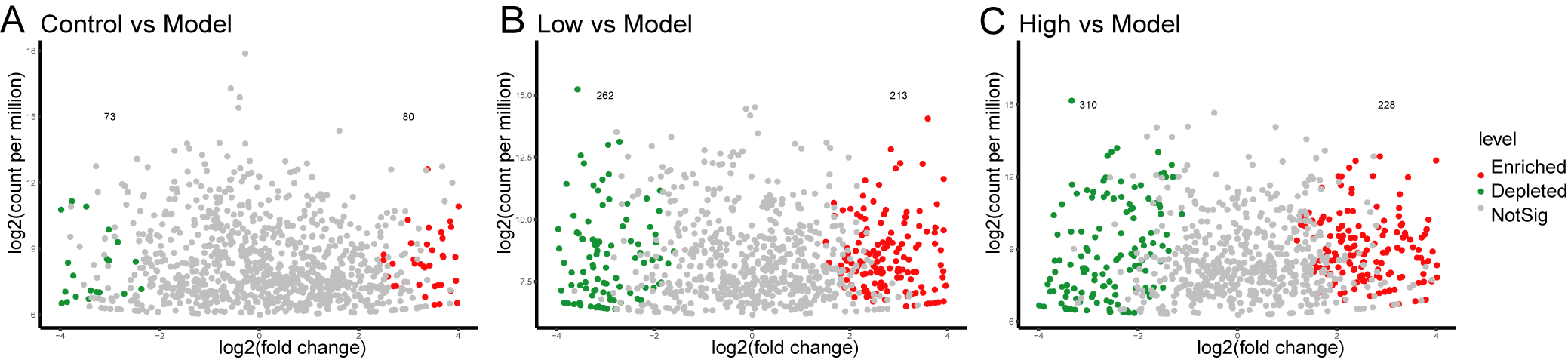

Supplement: FIGURE S2 — Differentially enriched and depleted OTUs between each group compared with the Model group. (A) Control vs Model; (B) Low vs Model; (C) High vs Model. Each point in the figure represents an individual OTU, of which the red points for significant enriched OTUs, green points for significant depleted OTUs, gray points for OTUs with no significant difference. The number labeled in the figure is the number of OTUs that significantly enriched or decreased. [file Image_2.TIF]
